# Pathological Roles of Pulmonary Cells in Acute Lung Injury: Lessons from Clinical Practice

**DOI:** 10.3390/ijms232315027

**Published:** 2022-11-30

**Authors:** Noriyuki Enomoto

**Affiliations:** 1 Second Division, Department of Internal Medicine, Hamamatsu University School of Medicine, Hamamatsu 431-3192, Japan; norieno@hama-med.ac.jp; Tel.: +81-53-435-2263; Fax: +81-53-435-2354; 2 Health Administration Center, Hamamatsu University School of Medicine, 1-20-1 Handayama, Hamamatsu 431-3192, Japan

**Keywords:** acute lung injury, acute exacerbation, idiopathic pulmonary fibrosis, anti-MDA5-antibody, CADM, EGFR-TKI, COVID-19

## Abstract

Interstitial lung diseases (ILD) are relatively rare and sometimes become life threatening. In particular, rapidly progressive ILD, which frequently presents as acute lung injury (ALI) on lung histopathology, shows poor prognosis if proper and immediate treatments are not initiated. These devastating conditions include acute exacerbation of idiopathic pulmonary fibrosis (AE-IPF), clinically amyopathic dermatomyositis (CADM), epidermal growth factor receptor-tyrosine kinase inhibitor (EGFR-TKI)-induced lung injury, and severe acute respiratory syndrome coronavirus 2 (SARS-CoV2) infection named coronavirus disease 2019 (COVID-19). In this review, clinical information, physical findings, laboratory examinations, and findings on lung high-resolution computed tomography and lung histopathology are presented, focusing on majorly damaged cells in each disease. Furthermore, treatments that should be immediately initiated in clinical practice for each disease are illustrated to save patients with these diseases.

## 1. Introduction

Interstitial lung disease (ILD) is a relatively rare pathological condition that can induce respiratory insufficiency. In particular, rapidly progressive ILD frequently causes acute respiratory failure and death. Notably, diffuse alveolar damage (DAD) pattern on HRCT or on lung histopathological specimens are tied to a poor prognosis [1,2,3,4]. This rapidly progressive ILD with a poor prognosis includes acute exacerbation of idiopathic pulmonary fibrosis (AE-IPF), clinically amyopathic dermatomyositis (CADM), epidermal growth factor receptor-tyrosine kinase inhibitor (EGFR-TKI)-induced lung injury, and severe acute respiratory syndrome coronavirus 2 (SARS-CoV2) infection, named coronavirus disease 2019 (COVID-19). These devastating diseases frequently cause acute lung injury (ALI), including DAD, although the damaged pulmonary cells, which sometimes become apoptotic, are quite different in each disease. In this review, we focus on the specific diseases that induce severe respiratory failure in clinical practice and the pathological roles of pulmonary cells in ALI.

## 2. Acute Exacerbation of IPF

**Figure 1:**
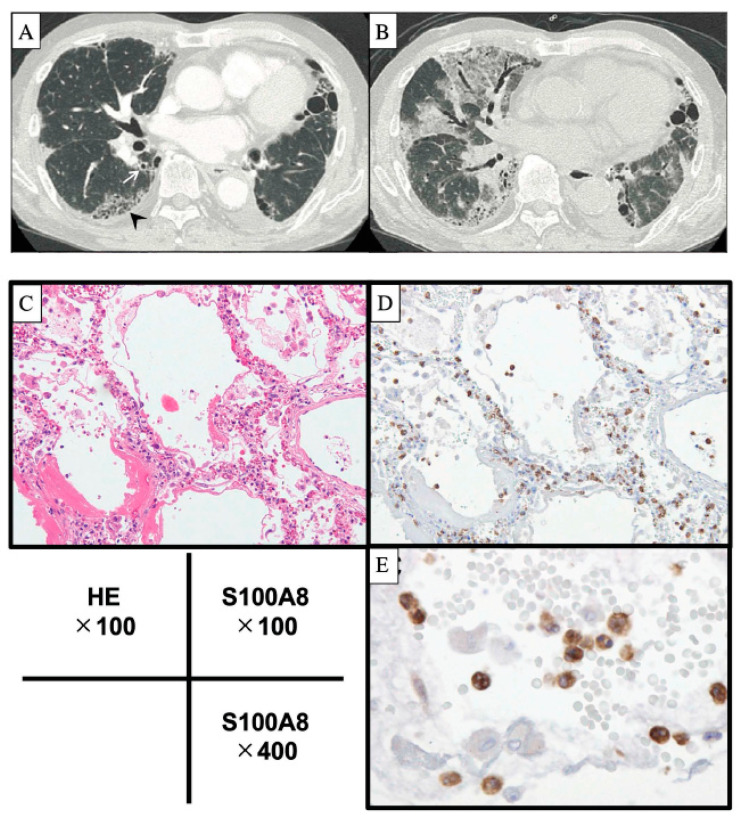
Findings on HRCT and histopathological specimens of the lungs in patients with AE-IPF. In a 78-year-old male patient with AE-IPF, HRCT at the initial diagnosis of IPF shows subpleural-predominant interstitial fibrosis, traction bronchiectasis (arrow) and honeycombing (arrowhead) ((**A**), from Oda et al. [1] with permission). HRCT at the onset of AE-IPF (12 months after the initial diagnosis) shows diffuse areas of GGA superimposed on underlying fibrotic opacities (**B**) [1]. Autopsy lung specimens were from another 52-year-old male patient with AE-IPF, who died on day 8 from the onset of AE- IPF. Hematoxylin and eosin staining shows diffuse alveolar damage with hyaline membrane ((**C**), from Tanaka et al. [2] with permission). Immunohistochemical staining of autopsy lung specimens shows that infiltrating alveolar neutrophils are positive for S100A8 (lower magnification in (**D**) and higher magnification in (**E**)), which is a calcium-binding protein produced and released mainly by activated neutrophils [2]. More neutrophils with S100A8 existed in the alveolar septa than in the alveolar space. Abbreviations: HRCT, high-resolution computed tomography; AE, acute exacerbation; IPF, idiopathic pulmonary fibrosis; GGA, ground-glass attenuation.

Acute exacerbation occurs within approximately one month of the clinical course of chronic progressive ILD, such as IPF [5,6,7]. Three cases of AE-IPF were first reported by Kondoh et al. in 1993 [8]. More AE-IPF occurs in Japanese patients than in Caucasian patients [9]. Therefore, racial and genetic predispositions to AE-IPF should exist in Japanese patients; however, this remains to be elucidated in detail. AE-IPF frequently occurs in elderly patients with end-stage IPF [10,11]. Baseline cardiovascular diseases and higher GAP stage (sex, age, physiology) were significant predictors of AE-IPF [10]. In patients with AE-IPF, lung ground-glass attenuation (GGA) and consolidation opacities appear on chronic fibrotic opacities on high-resolution computed tomography (HRCT) within approximately one month [5,7,12,13]. HRCT and histopathological findings of representative patients with AE-IPF are shown in Figure 1. The appearance of AE leads to poor prognosis in a staircase pattern [14] during the entire long-term course of IPF [15,16]. After the onset of AE, the 90-day mortality reached to 42.9–48.0% [17], and AE is one of the most frequent causes of death and accounts for 30–40% of the causes of deaths [18,19]. AE occurs more frequently in patients with IPF, at the rate of incidence 5.85–14.2%/person-year [15,16,20,21], than in those with other chronic ILD [21,22,23,24]. Patients with connective tissue diseases (CTD) experience AE-ILD at the rate of incidence 1.25-3.3%/person-year [22,23,24], and those with idiopathic non-specific interstitial pneumonia (iNSIP) experience AE-NSIP at the rate of incidence 4.2%/person-year [22]. Therefore, most of the knowledge of AE-ILD came from studies on AE-IPF because of the high frequency of AE-ILD in patients with IPF. With regard to CTD-ILD, AE-ILD most frequently occurs in patients with rheumatoid arthritis (RA) compared to other CTD [11] and has a poor prognosis after the onset of AE, similar to that in AE-IPF [11,24,25]. Furthermore, patients with unclassifiable IIP (UCIIP) also experience AE-UCIIP and show a poor prognosis after the onset of AE, similar to that in AE-IPF [21].

The diagnostic criteria for AE-IPF are as follows: (1) previous or concurrent diagnosis of IPF; (2) worsening of dyspnoea typically <1 month; (3) new bilateral GGA and/or consolidation on CT superimposed on the pattern of usual interstitial pneumonia (UIP); and (4) deterioration not fully explained by cardiac failure or fluid overload [17]. Although these criteria were prepared for AE of IPF, they seem to be applicable for other ILDs instead of IPF/UIP. Furthermore, AE-IPF consists of two subtypes: idiopathic and triggered AE, which include infection, post-operative AEs, drug toxicity, and aspiration [7]. The incidence of AE-IPF is high in winter [26,27]. Therefore, any respiratory infection may have contributed to the increased occurrence of AE-IPF. Torque teno virus was found by microarrays in patients with AE-IPF, and SARS-CoV2 infection was associated with a worse prognosis in those with AE-ILD than in AE-ILD without SARS-CoV2 infection [28]. Furthermore, vaccines against SARS-CoV2 reportedly may have induced AE-IPF [29], although the details remain to be elucidated. AE also occurs after events such as surgical operations [30], including surgical lung biopsy (SLB) [31,32] and bronchoalveolar lavage [33]. Even when a definite diagnosis of AE-IPF is not reached, suspected cases show poor prognosis, similar to definitively diagnosed patients with AE-IPF [27]. Therefore, patients with suspected AE-IPF should be immediately treated with steroids, similar to the treatment for AE-IPF, with concomitant administration of antibiotics for the treatment of causative conditions.

On HRCT, new lung GGA and consolidation opacities appear on chronic fibrotic opacities within approximately one month (Figure 1A,B) [1,5,7]. HRCT findings in AE-IPF are divided into three groups: peripheral, multifocal, and diffuse [12]. Diffuse pattern and large extent of alveolar opacity are significantly related to poor survival [13,34].

In lung histopathology, in addition to collagen deposition in the subpleural alveolar wall consistent with UIP, newly appeared DAD and neutrophil infiltration to the inner normal area are frequently seen in representative cases of AE-IPF (Figure 1C–E) [2,7]. In fact, in post-mortem autopsy specimens, a DAD pattern was observed in 78.8% of AE-IPF cases, while 28.8% exhibited pulmonary haemorrhage, and 17.3% developed thromboembolism [1]. Therefore, various histopathological findings exist in AE-IPF, and cautious diagnostic approaches are required for the histopathological diagnosis of AE-IPF when lung specimens are obtained, although surgical lung biopsy is not a prerequisite for its diagnosis.

As for pathogenesis of AE-IPF, related cells and molecules were summarized in Table 1. The levels of Krebs von den Lungen-6 (KL-6) [2,21], surfactant protein-D (SP-D) [2,21], α-defensin [35], and periostin [36,37] in the blood further increase in AE-IPF compared to those in the chronic phase, which are produced from damaged alveolar epithelial cells, and changes in KL-6 levels [38] or periostin [37] were significant prognostic factors in patients with AE-ILD. In addition, lactate dehydrogenase (LDH) levels increase in AE-IPF [2,21]. Activated neutrophils play an important role in the pathogenesis of AE-ILD/IPF [16,39,40,41], and the high number of neutrophils in bronchoalveolar lavage (BAL) is related to worse prognosis [40]. Furthermore, S100A8 (MRP8) and S100A9 (MRP14), calcium-binding proteins produced released mainly by activated neutrophils, are significant prognostic biomarkers (Figure 1D,E) [2]. Matrix metalloproteinase (MMP)-9, which is produced by activated neutrophils and facilitates the permeability of lung vessels, increases at AE-IPF [39]. In addition, interleukin-8 (IL-8) in BAL, which is related to the migration of neutrophils, also increases in AE-IPF [41]. Activated macrophages also play a role in AE-IPF pathogenesis. C-C motif chemokine ligand (CCL) 18, which is produced by activated macrophages and stimulates macrophages, known as profibrotic-M2 chemokine, increases in BAL in AE-IPF [41,42]. High levels of CCL18 in BAL [41] and higher hemosiderin scores of macrophages [43] are predictive for the development of future AE-ILD. Furthermore, activated macrophages produce ferritin, which is a significant prognostic factor of AE-IPF [44]. In the chronic stage of IPF, single-cell RNA-sequencing revealed an increased number of alveolar macrophages, dendritic cells (DC), and memory T-cells in the fibrotic lung [45]. In collaboration with profibrotic/M2 macrophages, CD4+helper T (Th)-cells such as Th2, Th17, and regulatory T-cell (Tregs) facilitate the profibrotic process [46]. These reports indicate that activated macrophages play a role in the pathogenesis of IPF, not only in the chronic stage, but also in AE-IPF. With regard to gastric aspiration and microorganisms in AE-IPF, higher levels of pepsin are found in BAL fluid in AE-IPF [47], and corisin peptide, which is derived from Staphylococcus and induces apoptosis of lung epithelial cells, is increased in both BAL fluid and serum in AE-IPF [48,49]. As for lung-protective molecules, the functions of heat shock protein (HSP) 70 and thrombomodulin have been reported. HSP70 is a functional TLR4 ligand that promotes endothelial cell survival during lethal oxidant injury [50]. Anti-HSP70 autoantibody was detected in 70% of patients with AE-IPF, while it was detected in 25% of those with IPF in chronic stage and 3% of healthy controls [51]. Thrombomodulin can bind to high-mobility group protein B1 (HMGB1) and prevent HMGB1 from binding to receptors for advanced glycation end-products (RAGE), consequently suppressing inflammation [52,53]. Thrombomodulin decreased [52,53], whereas HMGB1 increased [52,54,55] in AE-IPF. Adipocytes produce more adipokines in AE-IPF, such as adiponectin and leptin, and the adiponectin/leptin ratio is a significant prognostic marker for AE-IPF [56]. Other biomarkers for the prediction of AE-IPF appearance include a decrease of 10% or more in the percent predicted forced vital capacity (%FVC) within 6 months and baseline AaDO2 [57]. Other prognostic biomarkers for patients with AE-IPF include the baseline PaO2/FiO2 (P/F) ratio at AE [16,34], low baseline FVC and diffusion lung capacity for carbon monoxide (%DLCO) [26], C-reactive protein (CRP) [34], delay before initiating therapy [26], ΔP/F ratio 2 days after commencement of treatment for AE, and ΔLDH 2 days after commencement of AE treatment [58]. Recently, a risk-scoring system that predicts 3-month mortality has been reported [34]. This risk scoring system (PCR index) includes the P/F ratio, CRP level, and HRCT pattern, and this system could have well-segregated the prognosis of patients with AE-IPF [34].

As for treatments, AE-IPF may be treated with corticosteroids [59]; however, immunosuppressive therapy is often harmful in the chronic phase of IPF [60]. Steroid pulse therapy is frequently used as the initial therapy for AE-IPF, although evidence for this therapy is not strong enough. The addition of cyclophosphamide [25,61] or recombinant thrombomodulin [62] did not improve the survival of AE-IPF patients [62]. In contrast, a retrospective study showed that the use of antifibrotic agents (pirfenidone or nintedanib) improved the survival of patients with AE-IPF [63]. Furthermore, longitudinal use of nintedanib suppresses AE-IPF [64]. Therefore, antifibrotic agents should be a prerequisite underlying treatment for both acute and chronic stages in patients with IPF. As mentioned previously, activated neutrophils play an important role in the pathogenesis of AE-IPF [16,39,40]. Retrospective studies have reported that long-duration (6 h or more) direct hemoperfusion with a polymyxin B-immobilised fibre column (PMX-DHP) removed activated neutrophils [39] and significantly improved survival in patients with AE-IPF [58,65,66,67]. Furthermore, early commencement of long-duration PMX-DHP was effective in improving the survival of AE-IPF patients compared to later commencement of PMX-DHP [68,69]. PMX-DHP for AE-IPF is promising, but larger prospective studies are needed.

## 3. Clinically Amyopathic Dermatomyositis-Related ILD

Connective tissue diseases (CTD) sometimes show lung involvement such as ILD, pulmonary hypertension, bronchiolitis, bronchiectasis/bronchiolectasis, and serositis, including pleuritis/pericarditis, depending on the CTD [70]. These lung involvements lead to a wide variety of lung opacities on HRCT and histopathological findings in lung specimens. As for prognosis, particularly the presence of ILD or pulmonary hypertension, is associated with poor survival [71,72,73]. However, the prognosis of patients with CTD-ILD is significantly better than that of IPF patients [74]. CTD-related ILD is frequently seen in patients with systemic sclerosis (SSc) or in those with polymyositis (PM)/dermatomyositis (DM) [70], and ILD accounts for 35% of the causes of death in SSc [71], 48% in PM/DM [73], and 11% in rheumatoid arthritis (RA) [75]. PM/DM-ILD progresses more rapidly [76,77] than SSc-ILD [78] in many cases. Patients with PM/DM-ILD with acute/subacute onset show poorer survival than those with chronic onset [76,79,80]. In addition, DM-ILD shows worse survival than PM-ILD, and clinically amyopathic DM (CADM)-ILD has an even worse prognosis than DM-ILD or PM-ILD [77]. The definition of CADM includes both amyopathic and hypomyopathic DM [81]. Basic and clinical studies on disease-related antibodies have progressed in the field of myositis, and there are two major categories of autoantibodies: myositis-specific antibodies and myositis-associated antibodies [82]. The former includes anti-melanoma differentiation-associated gene (MDA) 5 antibody (previously called anti-CADM 140 antibody) and anti-amynoacyl-tRNA synthetase (ARS) antibodies. These two antibodies are exclusive and closely related to ILD rather than to myositis, although the prognoses differ widely among patients with each antibody. DM-ILD with anti-ARS antibody progresses slowly and responds well to corticosteroids; however, recurrence is not rare [80,83,84]. In contrast, DM-ILD with anti-MDA5 antibody progresses rapidly, is refractory to corticosteroid therapy, and shows poor survival [80,83,84,85,86]. MDA5 is a retinoic acid-inducible gene-I (RIG-I) family of intracellular viral sensors, and the positive rates of anti-MDA5 antibodies have been reported as 14.5–21.5% in DM [87,88] and 25% in PM/DM [80].

In patients with PM/DM and anti-MDA5 antibody, 53.3–82% of them have CADM [80,88,89,90], 94–95% have ILD [88,89], and 71–84% show rapidly progressive ILD [88] or acute/subacute (within 3 months) onset of ILD [80], which has a poor prognosis. Many patients with anti-MDA5 antibody live near the waterfront, and DM-ILD with anti-MDA5 antibody develops predominantly in October-March [90,91]. The exact reason for this is still unknown; however, MDA5 is known to recognise RNA viruses, and the induction of anti-MDA5 antibodies may be related to viral infection.

**Figure 2:**
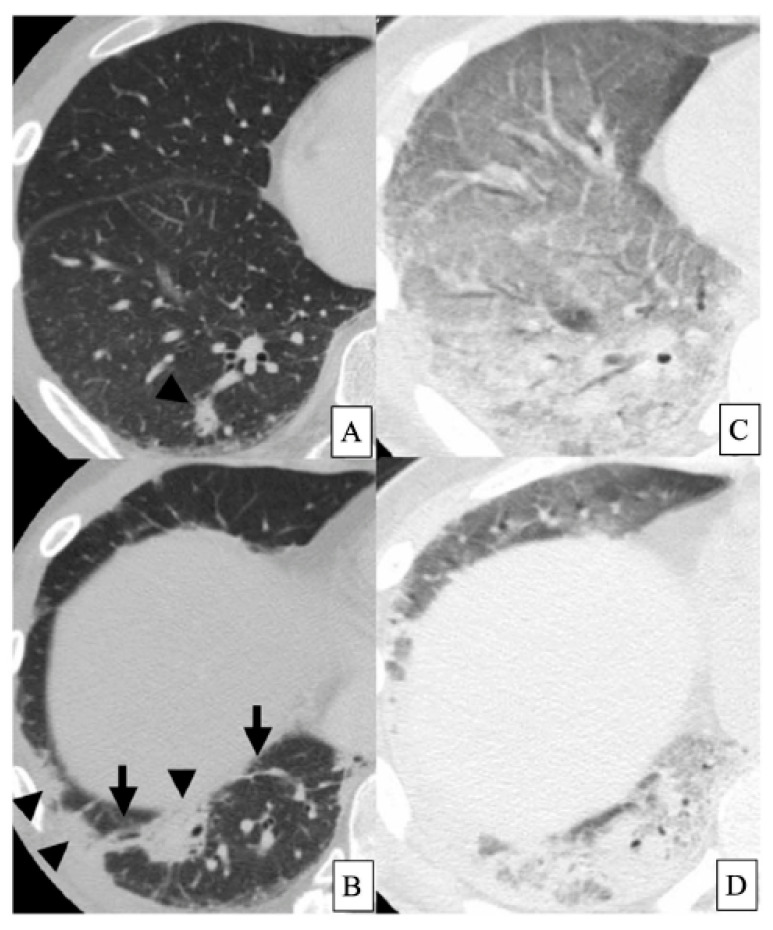
HRCT findings of the lungs in a patient with DM-ILD with anti-MDA5 antibody. A forty-four-year-old male patient with DM-ILD was positive for anti-MDA5 antibody. At diagnosis, peripheral and peribronchovascular consolidations are observed (arrowheads). Interlobular septal thickening and non-septal linear or plate-like opacities are also seen (arrows) ((**A**,**B**), from Tanizawa et al. [92] with permission). Despite treatments for 6 weeks, severe respiratory failure developed, requiring mechanical ventilation. Diffuse GGA and consolidation with air bronchograms are extended in the whole lungs (**C**,**D**) [92]. Surveillance at this point revealed no evidence of infection. The patient died of respiratory failure one week later. Abbreviations: HRCT, high-resolution computed tomography; DM, dermatomyositis; ILD, interstitial lung disease; MDA, anti-melanoma differentiation-associated gene; GGA, ground-glass attenuation.

HRCT findings of DM-ILD with anti-MDA5 antibody are mainly consolidation/GGA and random GGA in lower lung field, and these opacities occupied 83.3% of all cases [92] (Figure 2A,B). Furthermore, 60% of these patients with such opacities on HRCT died despite intensive immunosuppressive treatments [92] (Figure 2C,D). Similarly, the HRCT findings of anti-MDA5 antibody-positive cases are mainly consistent with the unclassifiable pattern of IIPs (66.7%) [80]. As for lung histopathological pattern, five of six cases of DM or CADM had a DAD pattern, and all these patients with DAD died of the progression of ILD [3]. Similarly, seven of nine patients with CADM-ILD with anti-MDA5 antibody had DAD in the lung histopathological specimens [93].

As for pathogenesis of CADM-ILD, related cells and molecules were summarized in Table 1. Activated macrophages play a key role in the pathogenesis of CADM-ILD with anti-MDA5 antibody. Patients with CADM-ILD and anti-MDA5 antibody exhibit activated alveolar macrophages producing ferritin, similar to cells in the bone marrow, liver, and spleen [94]. These findings look similar to macrophage activation syndrome with hyperferritinemic syndrome [95]. In DM-ILD, increased serum ferritin levels negatively correlate with the P/F ratio [96], and patients with higher ferritin levels show a significantly worse survival and serum ferritin level is a significant prognostic factor [96,97]. In addition, in patients with DM-ILD and anti-MDA5 antibody, high macrophage hemophagocytic scores are related to higher ferritin levels [98]. The serum level of CD206, which is preferentially expressed on the surface of alternatively activated (M2) macrophages, was significantly increased in patients with CADM/DM-ILD and anti-MDA5 antibody [97]. M2 macrophages are closely related to tissue repair and fibrosis and the interaction between M2 macrophages and alveolar epithelial cells may be crucial [99]. Similarly, CD163 is also expressed on alveolar macrophages, especially on M2 macrophages [100], and serum levels of soluble CD163 are significantly higher in patients with DM-ILD than in those with PM or without ILD [101]. Furthermore, serum chitotriosidase [102], which macrophages/neutrophils produce, and YKL-40 [103], which is a chitinase family and macrophages/epithelial cells produce, increased in PM/DM-ILD, especially in DM-ILD with anti-MDA5 antibody. As for prognosis related with above mentioned molecules, higher level of serum ferritin [96,97], CD206 [97], CD163 [104], chitotriosidase [102], YKL-40 [103], and hemophagocytic scores [98] are tied to a poor prognosis. Taken together, as indicated above, activated macrophages contribute to the pathophysiology of DM-ILD with anti-MDA5 antibody. The role of monocytes in this process has also been investigated. CCL2 and interferon-induced protein with tetratricopeptide repeats (IFIT) 3 mRNA expression in monocytes and serum CCL2 and interferon (IFN)-β levels are increased in patients with DM-ILD and anti-MDA5 antibody [105]. In addition, serum CCL2 levels are significantly higher in patients with DM-ILD and anti-MDA5 antibody [106]. Both macrophages and monocytes produce CCL2, and the migration of monocytes to the lungs, depending on CCL2, may play a role in the pathogenesis of DM-ILD with anti-MDA5 antibody. In contrast, low circulating lymphocytes and monocytes are found in patients with DM-ILD and anti-MDA5 antibody [107]. In addition, higher serum CCL2 levels [106] and the lower numbers of lymphocytes and monocytes in peripheral blood [107] are associated with a poor survival. The H-ferritin subunit can inhibit lymphoid and myeloid cell proliferation via the T-cell immunoglobulin and mucin domains (TIM) 2 [100,108]. Therefore, H-ferritin produced by macrophages may be related to lower lymphocyte and monocyte counts in blood in patients with DM-ILD and anti-MDA5 antibody. Other immune cells and cytokines, such as neutrophils [109] and IL-8 [109,110], are also associated with a poor prognosis in patients with DM-ILD and anti-MDA5 antibody.

In terms of therapy, when PM/DM-ILD is slowly progressive with chronic onset without anti-MDA5 or anti-ARS antibodies, corticosteroids alone may be sufficient for treatment. However, when anti-MDA5 or anti-ARS antibodies are positive or patients show acute/subacute onset, corticosteroids and calcineurin inhibitors (tacrolimus or cyclosporine A) should be administered [111,112]. Furthermore, patients with acute/subacute onset and anti-MDA5 antibody or patients with rapidly progressive ILD should be treated with corticosteroids, calcineurin inhibitors, and intravenous cyclophosphamide (IVCY) [113,114]. This triple combination therapy significantly improves survival compared to historical controls [115]. However, triple combination therapy significantly increases the incidence of serious infections [115], with an odds ratio of 5.51 [116]. Therefore, overtreatment should be avoided in DM-ILD patients without rapidly progressive ILD or anti-MDA5 antibody [113,114]. Moreover, if DM-ILD is refractory to the above-mentioned treatments, intravenous immunoglobulin (IVIG) should be considered [117,118]. Finally, when DM or CADM-ILD progress despite appropriate treatment, this type of ILD is recently called progressive fibrosing (PF)-ILD [119] or progressive pulmonary fibrosis (PPF) [59]. If progressive cases meet the criteria for PF-ILD or PPF, patients with DM or CADM-ILD should be treated with an anti-fibrotic agent (nintedanib) [119] following the above-mentioned immunosuppressive treatments.

## 4. EGFR-TKI-Induced Lung Injury

The number of patients with drug-induced lung injury (DLI) has increased with the development of new medicines. Especially in Japan, the number of DLI has strikingly increased since 2000 [120], and the representative causative drugs are EGFR-TKIs against non-small cell lung cancer (NSCLC), including gefitinib [4] and erlotinib [121], and antirheumatic drugs including leflunomide [122]. In the 2000s, severe DLI and death due to gefitinib were frequently reported [4] which was soon a serious public concern in Japan [120]. Gefitinib-induced lung injury was observed in 5.8% of Japanese patients with NSCLC, and the mortality rate was 38.6% [120]. The incidence of gefitinib-induced lung injury was higher than that of chemotherapy within the first four weeks (odds ratio, 3.2 [123]. Furthermore, erlotinib-induced lung injury was found in 4.3% of Japanese NSCLC patients, and the mortality rate was as high as 35.7% [121]. Risk factors for the development of EGFR-TKI-induced lung injury include the presence of comorbid ILD, smoking history, male sex, and performance status of two or more [120]. In particular, the presence of preceding ILD and smoking history are common risk factors for EGFR-TKIs [120] and leflunomide [122]. Moreover, the presence of preceding ILD was related to increased mortality in EGFR-TKI-induced lung injuries [123]. Therefore, screening for preceding ILD using chest HRCT before initiating EGFR-TKI therapy is important to prevent DLI and DLI-induced death in clinical practice. Japanese patients are predisposed to EGFR-TKI-induced lung injury compared to people of non-Japanese origin [124], and the odds ratio of all pneumonitis was 5.04 and that of grade 5 pneumonitis (death) was 4.55 [124]. Additionally, the frequency of EGFR-TKI-induced lung injury was not significantly different between Asian and non-Asian populations but was significantly higher in Japanese than in non-Japanese Asians (odds ratio 12.7) [125].

**Figure 3:**
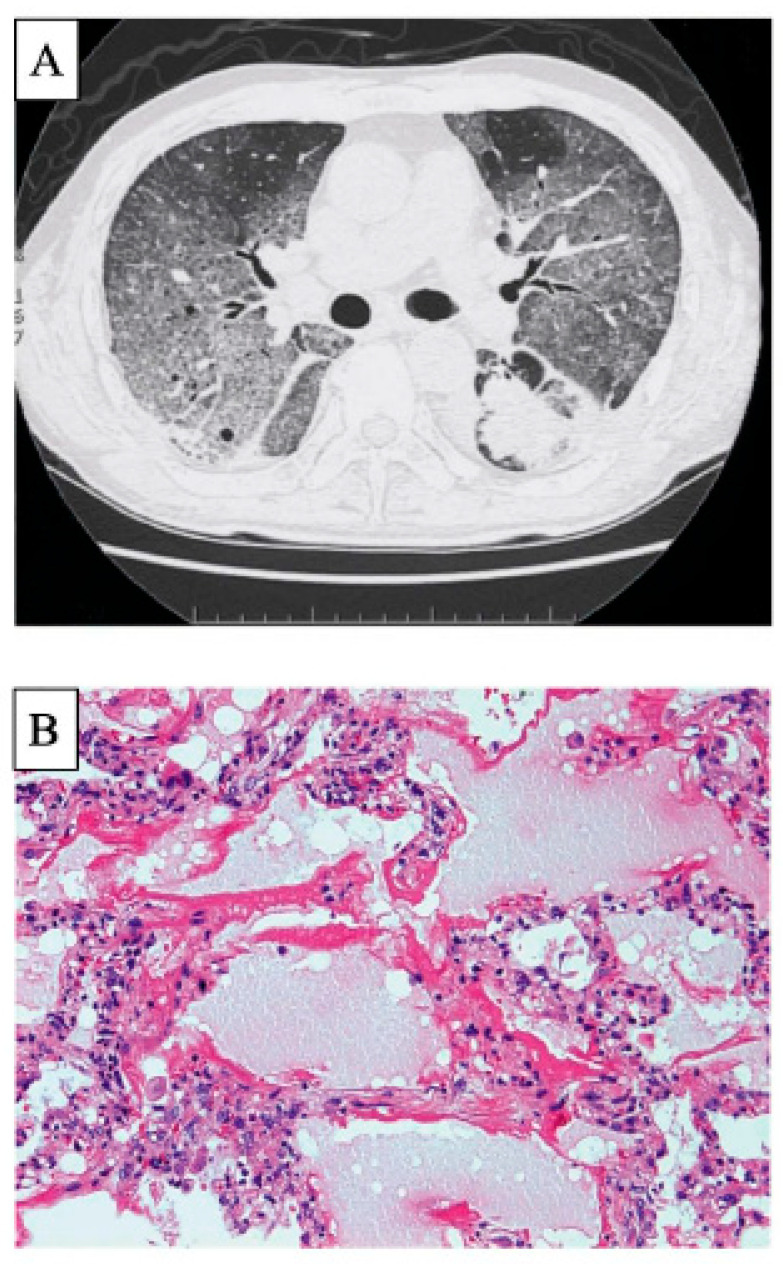
CT and histopathology of EGFR-TKI-induced lung injury. A 61-year-old male with lung adeno carcinoma received gefitinib. This case showed extensive diffuse GGA consistent with a DAD pattern on CT ((**A**), from Inoue et al. [4] with permission). Another 85-year-old male with lung squamous carcinoma and IPF received thoracic irradiation and gefitinib. A lung histopathological specimen with haematoxylin and eosin staining shows DAD pattern (**B**) [4]. This case died of rapidly progressive respiratory failure despite treatments with high-dose corticosteroids. Abbreviations: CT, computed tomography; EGFR-TKI, epidermal growth factor receptor-tyrosine kinase inhibitor; GGA, ground-glass attenuation; DAD, diffuse alveolar damage.

The HRCT patterns of DLI consist of five patterns: DAD, NSIP, organising pneumonia (OP), hypersensitivity pneumonitis (HP), and acute eosinophilic pneumonia (AEP) [126]. A CT image of the gefitinib-induced lung injury is shown in Figure 3A [4]. This case shows extensive diffuse GGA consistent with a DAD pattern. In this way, when patients show a DAD pattern on HRCT, they have the lowest P/F ratio, and the steroid cumulative dose is highest among these five patterns [126]. Patients with a DAD pattern on HRCT show significantly higher serum KL-6 levels, and those with more opacities on HRCT have higher serum KL-6 levels [127]. BAL in DAD includes many neutrophils [120]. In terms of EGFR-TKI-induced lung injury, patients frequently show DAD patterns on both HRCT (Figure 3A) and histopathology (Figure 3B), and many patients with a DAD pattern have a poor prognosis [4,120], with a mortality rate of approximately 40% [121]. Therefore, careful attention should be paid when patients with EGFR-TKI-induced lung injury exhibit a DAD pattern on HRCT. Moreover, infections such as pneumocystis pneumonia should be ruled out, especially when patients are immunocompromised or treated with corticosteroids, immunosuppressants, or biological agents [128].

In the pathogenesis of EGFR-TKI-induced lung injury, related cells and molecules were summarized in Table 1. Gefitinib induces lung inflammation via the production of IL-1β, and the release of HMGB1 from macrophages further leads to the death of macrophages [129,130]. This inflammation and subsequent programmed cell death is known as pyroptosis [129]. Gefitinib causes mitochondrial damage and mitochondrial reactive oxygen species (ROS) production. Mitochondrial ROS then activate the NLR family pyrin domain-containing protein (NLRP) 3 inflammasome and facilitate the production of IL-1β [129,130]. Furthermore, mitochondrial ROS induce DNA damage and facilitate the release of HMGB1, which can initiate a positive feedback loop of NLRP3 inflammasome signalling, leading to excessive inflammation [129,130]. In contrast, HSP70 protects lungs against pulmonary fibrosis, and administration of gefitinib suppresses the expression of HSP70 in the lungs and facilitates pulmonary fibrosis in a rodent model [131]. As written in the part of AE-ILD, HSP70 is a functionally TLR4 ligand during lethal oxidant injury that promotes endothelial cell survival [50]. Anti-HSP70 autoantibodies were detected in 70% of patients with AE-IPF compared to 3% of healthy controls [51]. Administration of geranylgeranylacetone, an inducer of HSP70, decreases lung inflammation and apoptosis of lung cells and further suppresses bleomycin-induced pulmonary fibrosis [132]. Furthermore, administration of geranylgeranylacetone suppresses gefitinib-induced exacerbation of pulmonary fibrosis [131]. Therefore, given these basic experimental results, the administration of NLR3-inhibitor, neutralising antibody of IL-1β, papaverine as an inhibitor of HMGB1 [130], and geranylgeranylacetone may be promising for the future treatment of EGFR-TKI-induced lung injury.

## 5. COVID-19 and ILD

**Figure 4:**
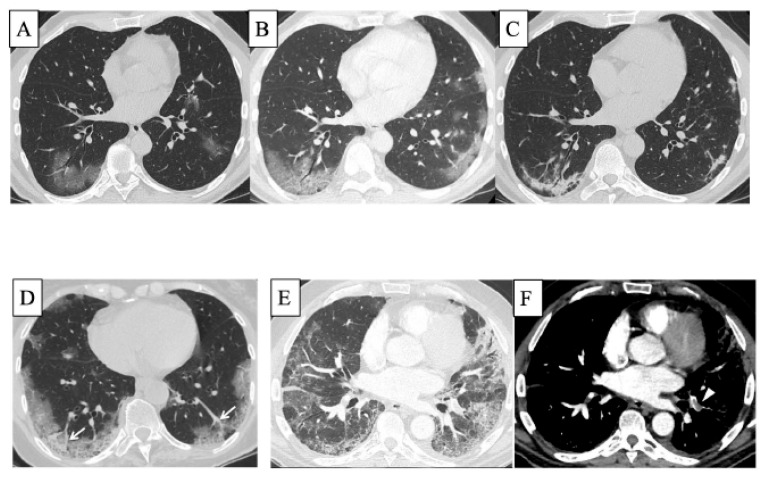
HRCT of the lungs in COVID-19. On HRCT, multiple GGA, crazy paving patterns, and consolidation are seen in peripheral or peribronchiolar lung area in many cases with COVID-19. These findings on HRCT change over time by phases, such as multiple GGA in the early phase (**A**), crazy paving appearance 10 days after the onset of symptoms in the progressive to peak phase (**B**), and multifocal consolidation with mild parenchymal distortion another 7 days later in absorption phase (**C**) (from Larici et al. [142] with permission). CT showing the presence of “enlarging vessel” sign within the areas of increased lung density (arrows, (**D**)) [142]. Acute pulmonary embolism is seen in severe COVID-19 patients (arrowhead, (**E**,**F**)) [142]. Abbreviations: HRCT, high-resolution computed tomography; COVID-19, coronavirus disease 2019; GGA, ground-glass attenuation.

SARS-CoV2 is an enveloped positive-sense single-stranded RNA virus. The SARS-CoV2 outbreak occurred in Wuhan at the end of 2019 and spread worldwide thereafter. This infection by SARS-CoV2 was named COVID-19 and was declared to be a pandemic in March 2020 by the World Health Organization (WHO). As of October 2022, more than 613 million people have been infected with SARS-CoV2 and more than 6.5 million deaths have occurred worldwide (information on WHO COVID-19 dashboard: https://covid19.who.int). COVID-19 is now the most frequent infectious disease in the world far exceeding the prevalence of tuberculosis. The excess mortality rate exceeded 300 deaths per 100,000 people in 21 countries from January 2020 to December 2021 [133]. Notably, the mortality risk was higher in the Delta variant pandemic period than in the Omicron variant period [134]. Although the clinical presentation of COVID-19 is highly variable, some patients experience hypoxaemia without discomfort, which is called “silent hypoxemia” [135]. Thereafter, in some cases, COVID-19 causes severe acute respiratory distress syndrome (ARDS), particularly in patients with risk factors such as older age, male sex, cardiovascular disease, chronic respiratory disease, diabetes, obesity, and hypertension [136,137]. Patients with pre-existing ILD, such as IPF, RA-ILD, or SSc-ILD, show worse survival than those without pre-existing ILD [138]. Furthermore, patients with COVID-19-related AE-ILD have a worse prognosis than those without COVID-19 [28]. In patients without pre-existing ILD, those with COVID-19 ARDS have a higher body mass index and longer duration of mechanical ventilation than those with non-COVID-19 ARDS; however, 60-day mortality is similar [139]. In addition, thromboses such as pulmonary embolism and venous thromboembolism, which may be related to hypoxia, occur more frequently in COVID-19 especially in severe COVID-19 cases than in other infectious pneumonia or in mild to moderate COVID-19 cases, with an incidence of 9.5–30% [140,141,142]. Microthrombi in capillaries are found in fatal COVID-19-associated lung injuries [143]. On HRCT, multiple GGA, crazy paving patterns, and consolidation are seen in the peripheral or peribronchiolar lung area in many cases with COVID-19 [142,144] (Figure 4). These findings on HRCT change over time by phases (Figure 4A, early phase; B, 10 days later; and C, another 7 days later) [142], and the more diffuse extent of the findings on CT is associated with higher severity of COVID-19 [142]. Moreover, pulmonary vessel enlargement is observed in or adjacent to GGA and consolidation [142,145] (Figure 4D). Hypoxic pulmonary vasoconstriction (HPV) may be impaired due to endothelial damage caused by SARS-CoV-2 infection and may be related to pulmonary vessel enlargement on HRCT [137,146]. In addition, as mentioned above, thromboses such as pulmonary embolism are seen in severe COVID-19 patients (Figure 4E,F) [142]. In autopsy lung specimens of patients with fatal COVID-19, DAD patterns with excessive thrombosis and injury to alveolar epithelial cells/endothelial cells have been observed [137,147,148].

SARS-CoV-2 invades human cells, including alveolar epithelial cells, vascular endothelial cells, and lymphocytes, via the angiotensin-converting enzyme (ACE) 2 receptor [149] and CD147 [150]. In the pathogenesis of COVID-19 and ILD, related cells and molecules were summarized in Table 1. Cellular senescence and mitochondrial dysfunction play important roles [149]. Mitochondrial dysfunction and apoptosis are observed in lymphocytes, especially T cells, which are important for protection from SARS-CoV-2 [151], and are related to lymphocytopenia in patients with COVID-19 [150,152,153]. Mitochondrial ROS and subsequent activation of the NLRP3 inflammasome appear to be related to severe respiratory failure in coronavirus infection [154,155]. Furthermore, abnormal mitochondrial ultrastructure and increased expression of inhibitory checkpoints, such as programmed death-1 (PD-1) and its ligand (PD-L1), are found in monocytes in patients with COVID-19 [156]; and non-classical monocytes are further decreased in those with severe COVID-19 [157]. In line with these reports, T cells also show higher levels of the exhausted marker PD-1 and reduced expression of CXCR6 [158], which is important for the localisation of resident memory T cells, and the number of CD4+ and CD8+ T cells is reduced [159,160]. Moreover, the reduction in CD4+ and CD8+ T cells is negatively correlated with survival in patients with COVID-19 [160]. Additionally, cytokine production and reactivation of SARS-CoV-2 specific CD8+ T cells are inhibited in severe COVID-19 cases [161]. Similarly, apoptosis in plasmacytoid dendritic cells (DC) [162] and the decreased number of plasmacytoid DC and myeloid DC are found in patients with COVID-19 [163], which may be related to the impaired protective function by type 1 IFN. Additionally, neutrophils accumulate in the lungs, and calprotectin (S100A8/S100A9), which is a calcium-binding protein mainly produced from activated neutrophils, promotes inflammation [164] and increases in blood in patients with severe COVID-19 [157]. Neutrophils and neutrophil extracellular traps (NETs) are abundantly present in seriously damaged COVID-19 lung tissue [143], similar to ALI in influenza pneumonitis [165]. Regarding lung cell death, SARS-CoV-2 proteins and induced cytokines lead to PANoptosis consisting of apoptosis, pyroptosis, and necroptosis in the same cell population, which is a unique inflammatory programmed cell death [155,166]. SARS-CoV-2-induced synergistic effect of tumor necrosis factor (TNF)-α and IFN-γ causes PANoptosis [166]. Additionally, SARS-CoV-2-induced activation of caspase-8 causes apoptosis [167]. In this pathway, phosphorylation of receptor-interacting protein kinase-3 (RIPK3) and mixed lineage kinase domain-like (MLKL) also induces necroptosis [167] and facilitates inflammation via IL-1β, which may be related to COVID-19-induced ARDS. In fact, serum level of RIPK3 is significantly higher in severe COVID-19 cases than in mild cases [168]. Furthermore, the open reading frame (ORF) 3a, a SARS-CoV-2 accessory viroprotein, induces apoptosis via activation of caspase-8 [169] or enhances pyroptosis of infected cells [155].

In terms of prophylaxis, newly developed messenger RNA (mRNA) vaccines not only significantly reduce the number of infected patients [170,171], but also decrease the severity of COVID-19, although the efficacy rates to prevent infection decreased in the Omicron variant pandemic period in 2022 [134,172]. As for treatments, early use of antiviral medicines and late use of corticosteroids seem to be beneficial [173]. As of October 2022, in the viral replication phase, starting nirmatrelvir/ritonavir [173,174] or Molnupiravir [175] within 3–5 days of symptom onset reduce the risk of hospitalisation or death. Baricitinib [176], a Janus kinase (JAK) inhibitor, or Baricitinib with remdesivir [177] reduces the time to recovery or 28-day mortality, however, remdesivir alone does not reduce 28-day mortality [178]. Monoclonal antibodies target spike proteins and decrease symptom duration and mortality against the Delta variant but not against the Omicron variant, except for sotrovimab [173]. In the inflammatory phase, 6 mg/day of dexamethasone for 10 days decreases 28-day mortality in hospitalised patients with severe COVID-19 [179], although a higher dose of methylprednisolone may also be effective. Tocilizumab [180], an IL-6 receptor antagonist, and prophylactic anticoagulation [173] are also effective in critically ill patients with COVID-19.

## 6. Conclusions

In this review, four diseases, AE of IPF, CADM, EGFR-TKI-induced lung injury, and COVID-19, which lead to rapid progressive ILD and respiratory failure, are reviewed. All these conditions have poor prognoses and frequently show ALI, including DAD, on HRCT and lung histopathological specimens. However, pathological cells in the lungs, which are mainly impacted and sometimes become apoptotic, are quite different in each disease. These cells include alveolar epithelial cells, pulmonary vascular endothelial cells, alveolar macrophages, lymphocytes, and neutrophils, which play important roles in the pathogenesis of various diseases. Therefore, attending doctors should carefully monitor patients with these diseases to check their clinical information, physical findings, laboratory examinations, and lung HRCT findings, and immediately initiate proper treatments to save these patients. Further novel medical developments that prevent and treat these diseases will be desired in the future.

## Figures and Tables

**Table 1:** Cells and molecules related with a pathogenesis of each disease.

	Alveolar Epithelial Cell Injury	Lung Endothelial Cell Injury	Activated Alveolar Macrophage	DC	Lymphocyte	Monocyte	Activated Neutrophil	Other Cells	Histopathological Patterns and Other Findings
**AE-IPF**	KL-6↑, SP-D↑ α-defensin↑ Periostin↑ Corisin↑ (staphylococcus) Pepsin↑, HMGB1↑ Apoptosis↑	HSP70↓ Thrombomodulin↓	CCL18↑ Ferritin↑ Hemosiderin score↑ HMGB1↑	DC↑	Memory T cells↑		S100A8↑ S100A9↑ MMP9↑ IL-8↑	Adiponectin↑ Leptin↑	DAD Pulmonary haemorrhage Thromboembolism
**CADM-ILD**	YKL-40↑		Anti-MDA5 ab↑ Ferritin↑, YKL-40↑ Chitotriosidase↑ CD163↑, CD206↑ CCL2↑ Interferon β↑ Hemophagocytic score↑		Lymphocyte↓ (H-Ferritin↑, TIM2)	Monocytes↓ (H-Ferritin↑, TIM2) CCL2↑ IFIT3↑	Chitotriosidase↑ IL-8↑		DAD
**EGFR-TKI-induced lung injury**		HSP70↓ Apoptosis↑	Mitochondrial ROS↑ NLRP3↑, IL-1β↑ HMGB1↑ Pyroptosis↑				Neutrophils↑		DAD
**COVID-19 and ILD**	Direct invasion of virus PANoptosis↑	Direct invasion of virus PANoptosis↑ Thrombosis↑		Plasmacytoid DC↓ Myeloid DC↓	Direct invasion of virus Lymphocytes↓ Mitochondrial ROS↑, NLRP3↑ PD-1↑, PDL-1↑ CXCR6↓, TNFα↑ IFNγ↑ PANoptosis↑	Monocytes↓ Mitochondrial damage↑ PD-1↑, PDL-1↑	S100A8↑ S100A9↑ NETs↑		DAD Thrombosis

Abbreviations: AE, acute exacerbation; IPF, idiopathic pulmonary fibrosis; CADM, clinically amyopathic dermatomyositis; ILD, interstitial lung disease; EGFR-TKI, epidermal growth factor receptor-tyrosine kinase inhibitor; COVID-19, coronavirus disease 2019; DC, dendritic cell; KL-6, Krebs von den Lungen-6; SP-D, surfactant protein-D; HMGB1, high mobility group box protein1; HSP, heat shock protein; MMP, matrix metalloproteinase; DAD, diffuse alveolar damage; TIM, T-cell immunoglobulin and mucin domains; IFIT, interferon-induced protein with tetratricopeptide repeats; ROS, reactive oxygen species; NLRP, NLR family pyrin domain-containing protein; NETs, neutrophil extracellular traps. “↑” means the increased concentration or numbers of the indicated molecules or cells. “↓” means the decreased concentration or numbers of the indicated molecules or cells.

## Data Availability

Not applicable.
